# Future directions for brain health clinics

**DOI:** 10.1192/bjo.2025.62

**Published:** 2025-05-09

**Authors:** Jerry H. K. Tan, Ivan Koychev

**Affiliations:** Department of Psychiatry, University of Oxford, Oxford, UK; Division of Psychiatry, Department of Brain Health Sciences, Imperial College London, London, UK

**Keywords:** Brain Health Services, memory clinic, mild cognitive impairment, biomarkers, Alzheimer’s disease

## Abstract

Brain Health Services are second-generation memory clinics that aim to reduce the risk of progression to dementia in at-risk individuals. We describe the rationale for such a service, and comment on its novel implementation by Venkataraman and colleagues that integrates digital technologies and biomarker testing. We describe the advantages and possible limitations of such an approach, then investigate areas for further work – namely, the need to account for multiple pathologies in biomarker testing and to formulate standards for genetic counselling.


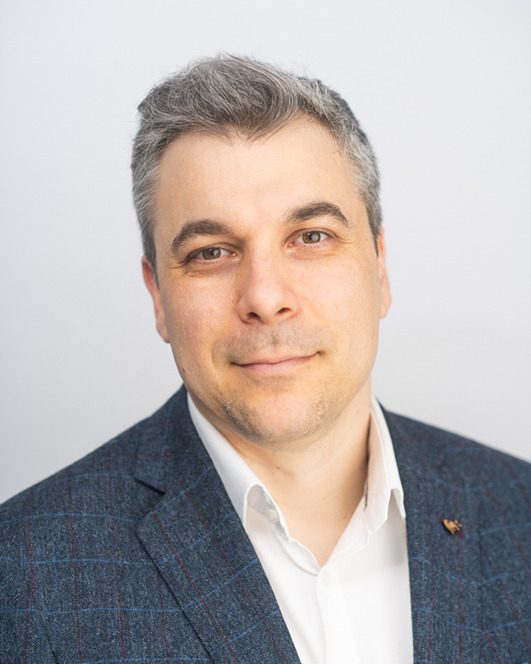
Memory services in the UK have undergone several key developments since they were first established in the 1980s.^1^ The approval of acetylcholinesterase inhibitors by the National Institute of Care Excellence (NICE) in 2001 led to the widespread prescription of symptomatic treatments for Alzheimer’s Dementia,^2^ and was followed by the approval of memantine several years later. The 2009 National Dementia Strategy prioritised raising awareness, early diagnosis and better care for people with dementia, and a subsequent improvement in national detection rates and reduced antipsychotic prescribing in dementia have since been observed.^3^ The intervening two decades have seen the development of radiotracer imaging and cerebrospinal fluid (CSF) biomarkers for amyloid and tau, which have been shown to increase diagnostic certainty and influence patient management in real-world memory clinic settings.^4,5^ While their initial remit of diagnosis and providing education to patients and their carers has remained, many memory services now also provide post-diagnostic support with a variety of pharmacological and non-pharmacological interventions.^6^ These aim to improve quality of life for patients and their carers, delay progression and manage the behavioural and psychological symptoms of dementia.

Our understanding of the relationship between ageing and cognition has also evolved. This is now seen as a spectrum, with normal cognition and frank dementia on opposite ends, with the intermediate categories of subjective cognitive decline (SCD) and mild cognitive impairment (MCI) lying between them. Not all individuals with SCD or MCI will progress to dementia; even in those that do progress, the underlying pathological substrate may not be Alzheimer’s disease but may instead be due to Lewy body disease, vascular infarcts, chronic traumatic encephalopathy or even a combination of pathological processes. Nevertheless, strong epidemiological evidence and multicentre randomised controlled trials have shown that multidomain lifestyle interventions have the potential to reduce the risk of progression to dementia in at-risk individuals.^7^ These have led to the development of second-generation memory clinics, termed Brain Health Services (BHS),^8^ to serve the needs of individuals at risk of developing dementia. The four main pillars of such a service are to (a) assess the risk of developing dementia in those with cognitive complaints and (b) communicate this risk, then to provide (c) personalised interventions for the prevention of dementia and (d) cognitive enhancement interventions where they are proven to be effective. This follows in the wake of a global push to prioritise brain health,^9^ and BHS also have the potential to indirectly improve outcomes for other neurological and psychiatric conditions through risk reduction, early diagnosis and referral to appropriate services.

In this issue, Venkataraman and colleagues describe a novel implementation of the BHS paradigm in a demographically diverse cohort using a digital service, South London and Maudsley (SLaM) National Health Service (NHS) Trust Brain Health Clinic (BHC).^10^ The authors report the feasibility and acceptability of this approach in a preliminary cohort of 68 individuals with MCI, SCD or mild dementia recruited from local memory services. Clinical assessment was conducted remotely via video conferencing or telephone and included a patient history, the completion of validated rating scales and a digital cognitive test. Biomarker assessments included magnetic resonance imaging, blood sampling, lumbar puncture and saliva genotyping. Of the subset of participants that underwent lumbar puncture for Alzheimer’s disease biomarkers, most had a Aβ42 result (65%) or pTau/Aβ42 ratio (55%) suggestive of Alzheimer’s pathology. A similar trend was seen in saliva genotyping, with 52% found to be ApoE4 carriers and 54% having a polygenic risk score suggestive of high risk of Alzheimer’s dementia progression. Based on these assessments, participants at high risk of progression to dementia were then offered online cognitive well-being or lifestyle intervention groups, in addition to being stratified for clinical trials. Responses from participants that completed feedback questionnaires were mostly positive.

This study demonstrates that the four pillars of BHS can be feasibly delivered in a safe and acceptable manner alongside existing NHS memory services. Furthermore, this is the first report of such a service being delivered remotely within the UK. The authors rightly point out that such an approach reduces some barriers to accessibility, has a lower carbon footprint and requires less clinical infrastructure compared existing services. Given the existing pressures on memory services and the fact that dementia is vastly underdiagnosed, remote delivery of services that allows for rapid scalability is required to meet unmet demand. Nevertheless, care must be taken to ensure that equity of access is not disrupted by digital exclusion. While most participants in the study completed assessments via virtual conferencing, a sizeable minority (27%) completed telephone assessments only, despite the availability of mitigating strategies. It is also striking that only 40% of participants found the technology used for remote appointments easy or very easy to use, with an even lower percentage (29%) reporting the digital cognitive assessment as easy or very easy to use. Low levels of digital literacy or difficulty accessing devices among some participants may account for this but, alternatively, early stages of neurodegeneration may negatively impact learning, impairing the acquisition of learning the new digital skills required for remote assessments. Moreover, a digital approach may not be suitable for more complex cases involving a language barrier or sensory impairments, and in-person assessments would allow for a wider range of adaptations to be made in such cases. As a result, BHS of the future are likely to utilise a hybrid model to account for these.

The SLaM BHC model also highlights the feasibility of implementing biomarker-based diagnosis of MCI alongside clinical assessment in a real-world memory clinic setting. Recent updates to the Alzheimer’s Association diagnostic criteria have redefined Alzheimer’s disease to be a biological entity characterised by Alzheimer’s dementia neuropathologic change, regardless of symptoms.^11^ As such, abnormal CSF biomarkers (Aβ 42/40, p-tau 181/Aβ 42 and t-tau/Aβ 42) are now considered diagnostic of Alzheimer’s dementia, even in individuals without dementia. However, CSF sampling is limited by the invasive nature of lumbar puncture and the need for specialist skills typically unavailable in memory clinic settings. The use of plasma biomarkers, chiefly p-tau217, overcomes these limitations. Recent work has shown that a commercially available p-tau217 assay had accuracy comparable to CSF biomarkers in predicted Alzheimer’s dementia neuropathology in cohorts both with and without cognitive impairment, and also outperformed other p-tau isotopes.^12^ The relative ease of blood sampling would also allow for deployment of testing at scale, thereby improving accessibility and facilitating earlier diagnosis. Further work is ongoing to validate these assays in real-world settings.

We also highlight two areas for further work in the implementation of BHS. First, there is a need to account for multiple pathologies in the aetiological diagnosis of cognitive impairment. While Alzheimer’s dementia is the most common cause of neurodegenerative dementia, in reality, multiple neurodegenerative pathologies can contribute to dementia. One large autopsy study found concomitant Lewy body disease in up to 55% of individuals with Alzheimer’s dementia, while 80% of individuals with Lewy body dementia had concomitant Alzheimer’s dementia pathology.^13^ This has important implications for prognostication, because individuals with multiple pathologies have been shown to have greater cognitive decline compared with those with a ‘pure’ neurodegenerative process. The presence of co-pathologies may also affect the accuracy of diagnostic biomarkers, and also affect response to treatment in clinical trials of disease-modifying drugs.^14^ Rather than dichotomising MCI into Alzheimer’s dementia positive and negative categories, an integrated approach could be adopted where individuals are screened for a variety of neurodegenerative pathologies (Alzheimer’s dementia neuropathological change, α-synuclein andTDP-43) then assigned an individual biomarker ‘fingerprint’ to inform personalised interventions for risk reduction and treatment.

Additionally, the ease of genetic testing with self-collected saliva samples, as evidenced by SLaM BHC, allows for genotype to be taken into account when assessing MCI. It is well established that the APOE4 allele increases the risk of developing Alzheimer’s dementia,^13^ and polygenic risk scores have been shown to discriminate between progressors and non-progressors in MCI.^15^ Because genetic material is inherently heritable, individuals assessed with genomic testing may want to know the implications of their results for their families. Since late-onset Alzheimer’s dementia is polygenically inherited and heavily influenced by environmental factors, counselling for such a condition is complex. Nevertheless, despite this complexity, no formal guidance for genetic counselling in MCI has been published on this in the past decade, irrespective of the nuances of explaining resulting in a knowledge gap for clinicians that urgently needs to be filled.

In summary, the development of brain health clinics represents a paradigm shift in how we think about cognitive impairment. Future advances in blood biomarkers, amyloid-targeting disease-modifying treatments and digital innovations will facilitate an in-depth assessment of risk and the formulation of a personalised package of interventions to reduce risk and delay progression to dementia in those with SCD or MCI.
